# Determinants of knowledge, attitudes and childbirth experiences related to obstetric violence among women who delivered in the public health facilities of Jimma Zone Southwest Ethiopia: A cross sectional study design

**DOI:** 10.1371/journal.pone.0347100

**Published:** 2026-04-16

**Authors:** Ayanos Taye, Tefera Belachew

**Affiliations:** 1 School of Nursing, Faculty of Health Science, Institute of Health, Jimma University, Oromia, Ethiopia; 2 Department of Nutrition and Dietetics, Faculty of Public Health, Institute of Health, Jimma University, Oromia, Ethiopia; Federal Medical Centre Birnin Kudu, NIGERIA

## Abstract

**Background:**

The World Health Organization recognizes every woman has the right to respectful and dignified care during childbirth. However, a significant number of women experienced obstetric violence (OV) acts or omissions causing harm, violating rights, or undermining autonomy, remains prevalent globally, including in Ethiopia where women’s knowledge gaps and unfavorable attitudes perpetuate it due to limited rights awareness and cultural norms. In southwest Ethiopia, evidence on women’s knowledge, attitudes, childbirth experience related to OV, and their determinants among facility-based deliveries is scarce. This study thus investigates these factors among mothers delivering in public health facilities.

**Method:**

An institutional-based retrospective cross-sectional study was conducted among 402 women in public hospitals of the Jimma zone from August 12 to October 12, 2022. A simple random sampling technique was used to select the study subjects for structured, pre-tested face-to-face exit interviews. Data were entered into Epi-Data version 3.1 and exported to SPSS version 23 for analysis. Then, factors that showed significant associations in a bivariable linear regression model were added to multivariable linear regression models. Variables that had a P-value of < 0.05 in the multivariable model were considered statistically significant. Finaly, the results were presented using texts, tables and graphs.

**Results:**

The present study showed that more than two-thirds of women had a knowledge about universal rights during childbirth with a mean score of 14.89 ± 6.76, 68). About two out of three participants had unfavorable attitude towards childbirth experience (mean 33.55 ± 4.91, 66%) and experienced OV (mean 67.09 ± 11.79, 64%) during labor and childbirth.

Women living in urban areas (β = −4.69, 95% CI: −6.41, −2.96), those with antenatal care (ANC) contacts (β = −9.65, 95% CI: −11.18, −8.11), who gave birth at night (β = 4.06, 95% CI: 2.42, 5.70), and without obstetric complications (β = −8.07, 95% CI: −9.98, −6.16) showed significant associations with obstetric violence.

ANC contacts (β = 3.23, 95% CI: 2.02, 4.45) and companion utilization (β = 4.87, 95% CI: 2.14, 7.59) positively associated with women’s knowledge levels.

Absence of obstetric complications (β = −3.06, 95% CI: −4.00, −2.11), urban residence (β = −1.89, 95% CI: (−2.86, −1.01) and ANC contacts (β = −3.36, 95% CI: −4.13, −2.59) were associated with unfavorable attitudes toward childbirth experience related to obstetric violence.

**Conclusion:**

The study revealed that more than two-thirds of participants had relatively lower knowledge of childbirth rights. Likewise, approximately two-thirds held unfavorable attitudes and experienced obstetric violence (OV). The most commonly reported forms OV were non-dignified care, non-consented care, and physical abuse. Sociodemographic factors (residence, maternal occupation) and maternal factors (ANC contacts, obstetric complications, companion utilization) associated with women’s knowledge, attitudes, and childbirth experience related to OV.

To mitigate the challenges, prioritize comprehensive training for providers on respectful and dignified care, alongside women’s education to foster rights awareness and self-advocacy. Enhance ANC access, companion policies, and targeted strategies for high-risk groups (e.g., rural residents, certain occupations) to create supportive childbirth environments.

## Introduction

Obstetric violence (OV) is an alarming issue that affects women’s rights and health outcomes globally, particularly in low- and middle-income countries. The World Health Organization (WHO) recognizes that every woman has the right to receive respectful and dignified care during pregnancy and childbirth [1]. However, reports from various countries indicate that OV remains prevalent, with women experiencing various forms of disrespect and abusive practices, including non-consented medical procedures, non-dignified care, physical and verbal abuse, and neglect. Studies reveal that a significant proportion of women experience some form of OV during labor and delivery, with prevalence rates varying widely across different regions. For instance, evidences indicated that between 49.5% to 98% of women reported experiencing OV in various contexts, highlighting the urgent need for interventions to address this pervasive issue [2–5]

The severity of OV can have profound implications for women’s physical and mental health. Women who experience mistreatment during childbirth are at increased risk of complications such as postpartum depression, anxiety, and trauma. The psychological impact can deter women from seeking necessary healthcare services in the future, perpetuating cycles of poor maternal and child health outcomes. In many cases, the normalization of violence within healthcare settings contributes to the high prevalence of OV, as seen in studies from several African countries where cultural acceptance of such behaviors is prevalent [6–8]. This normalization not only affects individual experiences but also undermines trust in healthcare systems.

In Ethiopia and other African countries, the impacts of OV extend beyond individual women to affect families and communities at large. Women who experience mistreatment during childbirth may develop distrust towards healthcare providers, leading to decreased utilization of maternal health services. This reluctance can contribute to higher maternal and infant mortality rates, as women may avoid seeking care during subsequent pregnancies or for their children due to fear of mistreatment [2,9,10].

Women’s knowledge of their rights during childbirth plays a crucial role in addressing obstetric violence, which encompasses mistreatment, abuse, and non-consensual procedures in healthcare settings. Studies indicate that many women lack comprehensive awareness of their reproductive rights, leading to increased vulnerability during labor and delivery [11]. This gap in knowledge often stems from systemic factors within healthcare systems, including inadequate education and cultural norms that normalize certain forms of disrespect. For instance, research highlights that obstetric violence is recognized as a human rights violation, yet women’s understanding of these rights remains limited in many regions, perpetuating cycles of abuse [11,12].

Women’s attitudes towards obstetric violence are often shaped by cultural, societal, and patriarchal influences that lead to its internalization and acceptance as a routine aspect of childbirth, hindering recognition and advocacy for respectful maternity care. Many women perceive such mistreatment not as violence but as standard medical practice, resulting in psychological trauma, postpartum depression, post-traumatic stress disorder, and reluctance to seek future healthcare, which perpetuates cycles of poor maternal outcomes and erodes trust in health systems. This attitudinal barrier, compounded by stigma and power imbalances, underscores the urgent need for awareness campaigns and policy reforms to empower women to identify and challenge obstetric violence, ensuring equitable and rights-based reproductive health services [12–15].

Numerous studies have identified key factors contributing to obstetric violence, women’s knowledge, and attitudes during childbirth experiences in Ethiopia. These include socio-cultural beliefs surrounding childbirth, inadequate training among healthcare providers, and systemic barriers within the healthcare system. For instance, women who experience obstetric violence during delivery are less likely to use health facilities for future births, while mothers with greater knowledge of their maternity care rights encounter fewer instances of such violence [16–18]. Additionally, women’s attitudes toward facility-based childbirth play a significant role; those with negative perceptions are more likely to face mistreatment during labor and childbirth [19].

In Ethiopia, particularly southwest Ethiopia, evidence remains scarce on women’s awareness of their rights during intrapartum care, their attitudes, and experiences of childbirth related to obstetric violence. Therefore, this study aimed to identify determinants of women knowledge, attitude and childbirth experience related obstetric violence in public health facilities of southwest Ethiopia.

## Methods and materials

### Study design, setting and population

A facility based cross-sectional study with quantitative approach was employed in public hospitals in the Jimma zone Southwest Ethiopia from August 12,2022 to October 12, 2022 to recruit study participants. Cross-sectional study design captures data on exposures and outcomes simultaneously from a population sample at one point in time, providing a snapshot ideal for assessing prevalence in maternal health research, such as women’s knowledge, attitude and obstetric violence in Ethiopia. Participants qualify via inclusion criteria without prior selection by exposure or outcome, enabling analysis of multiple variables Jimma Zone is one of the zones of Oromia Regional State, which has an estimated total population of more than 3,538,463. Of these, males account for 1,779,834 and females1,758,629. In Jimma Zone, there is one Tertiary hospital, two secondary hospitals, four primary hospitals, 115 health centers and 520 health posts. These hospitals namely include Jimma Medical Center, Shenen Gibe Hospital, Limmu Hospital, Agaro Hospital, Dimitu Hospital, Seka Chokorsa Hospital and Nada Hospital. For this study, Seka Hospital(primary) and Agaro Hospital(secondary) were selected. In both hospitals, different health services including Maternal and Child Health (MCH) services are provided. Seka Hospital is one of the zonal hospitals which is serving more than 2 million populations per year. The Seka hospital is accountable to the Seka Woreda Health Office whereas Agaro Hospital is accountable to the Agaro Town Health office. The two hospitals are 60 km away from each other. The monthly Antenatal Care (ANC), institution delivery and Postnatal Care (PNC) coverage in both study areas is 350, 432 and 456, respectively. The number of staff in Seka Hospital &Agaro Hospital is 32 & 27, respectively [20]. The number of doctors and midwives in both hospitals are 11 and 23, respectively at the time of data collection.

#### Study participants.

The target population for this study was postpartum women. Women who delivered in the study settings at term, had a vaginal delivery, and provided informed consent were included in the study, whereas those who underwent cesarean section or experienced severe medical or obstetric complications were excluded to ensure alignment with the study objectives Approximately five women refused to participate or discontinued the interview due to illness

### Sample size and sampling procedure

The sample size was determined using a single population proportion formula based on the following assumptions: the proportion of mothers who experienced disrespect and abuse in service delivery points of 49.7%) [2], 95% level of confidence level, 0.05 margin of error between the sample and the population and 6% non-retrieval rate. Therefore, the final sample size for the study was 407.

Two hospitals, Agaro Hospital and Seka Hospital, were purposively selected from the six public hospitals in the Jimma zone Southwest Ethiopia. Selection criteria included the annual volume of women’s deliveries, facility type, and comparable distribution of healthcare providers. Jimma University Medical Center and the remaining hospitals were excluded due to factors such as high student turnover for clinical practice, greater distance from the central city, impacts of the COVID-19 pandemic, low patient flow, and security or stability concerns.. In each selected facility, a sampling frame was developed by compiling a list of all postpartum women attending during the study period, sourced primarily from medical records or appointment logs. To achieve randomness, simple random sampling was applied: each woman on the list was assigned a unique identifier, and participants were selected using a lotter method until the target sample size was met. The number of women from each selected public hospitals was determined by proportionally allocating to the calculated 407 sample size to the total number of women in 2 months.

### Operational definition

Obstetric violence is a violation of human rights, emphasizing disrespect, abuse, and neglect in maternity care settings [21]. It was measured via the Bowers and Hills categories to measure disrespect and abuse, which was first developed by Shiferaw et al. (2016) in Ethiopia, demonstrating its reliability (α = 0.845). This instrument consists of six domains and 29 items, including nonconfidential care (4 items), non-consented care (4 items), non-dignified care (10 items), discriminatory care (4 items), detention in health facilities (2 items) and physical abuse (5 items). A five-point Likert scale ranging from “never: 1” to “always: 5” was used. The overall OV score was calculated by summing the items. Statements with negative conceptions were scored negatively. High composite scores on this scale indicate that participants experienced OV during childbirth (Table 1).

**Table 1:** Domains of obstetric violence manifestations.

Obstetric violence domains	Manifestations/specifications
Non-confidential care	Sharing private information the women, exposing the mother’s body during childbirth, and lack of visual barriers (such as cloths, blankets, or screens) during delivery.
Non-dignified care	Shouting or scolding, unfriendly welcoming, disrespectful treatment, negative or disparaging comment about women, Use of language that is not easily understood by the women, and failure to inform the women about alternative birth positions.
Non-consented care	Failing to provide information before and during procedures, neglecting to obtain the mother’s consent beforehand, and not encouraging women to ask questions about their care.
Physical Abuse	Hitting, slapping, pushing, pinching, pushed, tied to delivery bed, restraining, and Failure to adequately manage the mother’s pain.
Discrimination	Mistreating women based on social class, poverty, ethnicity, religion, tribe, age, marital status, or health status. Leaving women alone during labour and prohibiting the presence of a companion during labour and childbirth.
Detention	Unnecessary detention of women in health facilities due to inability to pay medical expenses and other reasons.

**Knowledge assessment:** Women’s knowledge was assessed using a 22-item questionnaire administered to the study participants in the selected health facilities. Responses included “yes,” “no,” or “don’t know,” with “yes” coded as ‘1’ and “no” or “don’t know” coded as ‘0.’ The total score for each construct was computed by summing raw scores from each question, and the mean was calculated. Higher composite scores indicated greater knowledge. The reliability of the items was evaluated using Cronbach’s alpha, yielding a value of 0.949, which demonstrates excellent internal consistency.

**Attitude assessment:** Women’s attitudes were assessed using a 13-item questionnaire with a five-point Likert scale ranging from “strongly agree” (scored as 5) to “strongly disagree” (scored as 1). All statements were negatively framed, meaning higher scores reflected stronger agreement with negative attitudes. The attitude scale ranged from a minimum score of 13 to a maximum of 65. The total score was computed by summing the raw scores from each item, and the mean was calculated. Higher composite scores indicated more unfavorable attitudes toward obstetric violence. However, in this study, a decrease in composite scores over time suggested a reduction in unfavorable attitudes, reflecting positive change.

The wealth index is measured using 18 survey questions about household assets, which was developed by Filmer and Prichett [22] Thus, a wealth index indicator variable was created to represent socio-economic status based on questions assessing a woman’s household ownership of specific items (radio, television, bicycle, phone, refrigerator, scooter, automobile) as well as household characteristics (flooring and roofing materials, water sources, toilet facilities, electricity). Principal Component Analysis (PCA) was employed to construct wealth indices by analyzing household asset data. The wealth index was generated using the final asset scores, which were based on an analysis of the whole sample to poorest, second, middle, fourth and richest

Housewife: Traditionally gendered term for a married woman whose primary role is unpaid home care, often carrying cultural connotations of domesticity without external employment.

Rural: Women who reside in non-urban (rural) areas, typically beyond the administrative boundaries of urban centers

### Data collection and measurement

Data collection instruments were adapted from validated surveys used in prior research in Kenya and Tanzania, as well as the Maternal and Child Survival Programs (MCSP) [21,23–26]

A language specialist first translated the questions into the local languages (Afan Oromo and Amharic) spoken in the study area. The translated items were then back-translated into English to ensure consistency in meaning and intent.

Data were collected through face-to-face exit interviews to assess women’s socio-demographic and economic characteristics, maternal-related factors, perceptions (women’s knowledge and attitudes) and obstetric violence related issues. Four female data collectors and two supervisors conducted the process over two months in the study areas. Data collectors held at least a BSc, MSc, or MPH in health sciences or sociology and were fluent in Afan Oromo and Amharic to ensure effective communication. Supervisors oversaw the process and addressed issues faced by the research assistants. Prior to actual data collection, two enumerators pretested the questionnaire on 5% of the sample size (35 women) at Limmu Hospital, 35 km from the study area. This step evaluated clarity, timing, and cultural fit for study items.

To ensure high-quality data collection, data collectors and supervisors received three days of training on interview techniques. Cronbach’s alpha scores were calculated for the knowledge and attitude scales to assess their reliability, yielding excellent reliability at 0.960 and 0.788, respectively. Content validity was established through review by three maternal health experts, who evaluated item relevance and comprehensiveness. Face validity was confirmed by these experts, supported by participant feedback from a pretest involving 5% of the target population (35 women). The pretest identified ambiguities and confirmed question clarity.

### Data management and statistical analysis

Data were entered twice into EpiData version 3.1 for accuracy, then exported to SPSS version 23 for analysis, with errors corrected by cross-checking original questionnaires. Descriptive statistics summarized sociodemographic, economic, maternal characteristics, knowledge, attitudes, and obstetric violence variables. Principal component analysis (PCA) computed wealth index for economic status after verifying assumptions of linearity and correlation. Simple linear regression identified candidate predictors by examining bivariate associations between individual exposures and continuous outcomes (knowledge/attitude/obstetric violence scores) at P < 0.25, screening variables for multivariable entry. These candidate variables entered multivariable linear regression via the enter method to adjust for confounders. All assumptions of normality of residuals and homoscedasticity were verified prior to analysis. Multicollinearity was also not detected, as all variance inflation factor (VIF) values were below 10 (maximum VIF = 1.8), which are performed following bivariate analysis. Adjusted unstandardized beta coefficients (β) with 95% confidence intervals were used to quantify independent associations, with statistical significance set at P < 0.05. Results were presented in tables, graphs, and text comprehensively.

### Ethical and environmental considerations

Ethical clearance (Ref. No. IRB/000/200/2020) was obtained from Jimma University Institutional Review Board (IRB) prior to data collection. Prospective participants received comprehensive information about the study’s purpose, procedures, benefits, risks, and confidentiality measures to enable informed decision-making.

Written informed consent was secured from all participants after they voluntarily agreed to join, affirming their autonomy through uncoerced choices. Participants were explicitly informed that involvement was voluntary, with no penalties, sanctions, or special remuneration, and they could withdraw at any time without providing a reason. To ensure participant privacy, interviews were conducted in a private room.

## Results

### Sociodemographic characteristics

Among 407 questionnaires administered, a total of 402 postnatal women were interviewed and completed the survey, with an overall average response rate of 98.8%.

The mean age of the women was 25.41 SD ± 4.89 years old. The majority 282(70.1%) of the respondents were between 20–34 years old, followed by age group 19–20, 99(24.6%). About 70.1% of the study participants were rural residents and more than eighty percent 329(81.8%) of women were Muslim by religion. Almost all respondents 395(98.3%) were married and greater than two out of five 165(41%) of the study participants attended primary education. Regarding the occupational status of the women, more than three out of five 315(78.4%) were housewives. As to the wealth status of the women, a majority 20.90% of them were in the poorest category of wealth quantile, which was followed by fourth, accounting for 19.65% (Table 2).

**Table 2:** Socio-demographic characteristics of women in Jimma Zone public hospitals, South West Ethiopia, 2023 (n = 402).

Variable	Category	Seka Hospital		Agaro Hospital		Total	
n	%	n	%	n	%
Age(year)	19-20	62	30.7	37	18.5	99	24.6
	20-34	132	65.3	150	75.0	282	70.1
	35-49	8	4.0	13	6.5	21	5.2
Mean Age 24.35 ± 4.58 26.39 ± 5.15 25.41 SD ± 4.89							
Residence	Rural	120	59.4	107	53.5	227	56.5
	Urban	82	40.6	93	46.5	175	43.5
Religion	Muslim	155	76.7	174	87.0	329	81.8
	Orthodox	35	17.3	19	9.5	54	13.4
	Protestant	10	5.0	6	3.0	16	4.0
	Other (pagan and Jehova)	2	1.0	1	.5	3	0.7
Marital status	Never married	2	1.0	3	1.5	5	1.2
	Married	199	98.5	196	98.0	395	98.3
	Divorced	1	.5	1	.5	2	0.5
Educational status	No education	75	37.1	38	19.0	113	28.1
	Primary	84	41.6	81	40.5	165	41.0
	Secondary	32	15.8	60	30.0	92	22.9
	More than secondary	11	5.4	21	10.5	32	8.0
Occupational status	Housewife	135	66.8	180	90.0	315	78.4
	Farmer	20	9.9	2	1.0	22	5.5
	Business	20	9.9	3	1.5	23	5.7
	Student	9	4.5	3	1.5	12	3.0
	Employee	18	8.9	12	6.0	30	7.5
Wealth	Poor	27	13.4	57	28.5	84	20.9
	Second	27	13.4	50	25.0	77	19.2
	Middle	36	17.8	44	22.0	80	19.9
	Fourth	50	24.8	32	16.0	82	20.4
	Richest	62	30.7	17	8.5	79	19.7

Other: Pagan, Jehovah.

**Maternal characteristics.** All women who underwent the study research had received Antenatal care (ANC). Among women who received ANC, 291(72.4%) of them visited health facilities 2–3 times during their entire pregnancy. The median ANC contact was three. Hundred fifty (37.3%) of the study subjects had a parity of 2–3. Greater than two-fifths (41.5%) of the women reported the distance from home to the health facility location to be not long at all. Among the women who gave birth in the study facilities, 244(60.7%) of deliveries were conducted by males,76(18.9%) births were on Sunday and the majority 235(58.5%) of births were conducted at nighttime. All births were conducted by obstetric healthcare providers (Midwives and obstetricians). Regarding mother complications, 287(71.4%) of the women had not experienced any type of obstetrics complications during or after childbirth but approximately three out of ten (28.6%) mothers developed complications that arise during the intrapartum and postpartum period. Regarding the intention of women on birth, most of the women 340(84.6%) had a plan to have more children. Nearly half of participants (180, 44.8%) planned to deliver at the current facility. According to the report from women, the majority of disrespect and abuse the women experienced during labour and childbirth were committed by female health care providers, 148(36.8%). Concerning companion utilization, a greater number of women 379(94.3%) were not allowed to have a companion during childbirth in the delivery room (Table 3).

**Table 3:** Obstetric characteristics of women in Jimma Zone public hospitals, South West Ethiopia, 2023 (n = 402).

Variable	Category	Frequency	Percent
No. of ANC contact	1	54	13.4
	2-3	291	72.4
	>=4	57	14.2
Median ANC contact	3		
Parity (Birth order)	1	150	37.3
	2-3	150	37.3
	4-5	68	16.9
	>6	34	8.5
Sex of health care provider conducted delivery	Male	244	60.7
	Female	158	39.3
Day of the week mother gave birth	Sunday	76	18.9
	Monday	67	16.7
	Tuesday	62	15.4
	Wednesday	60	14.9
	Thursday	48	11.9
	Friday	48	11.9
	Saturday	41	10.2
Time of birth	Day time	167	41.5
	Nighttime	235	58.5
Obstetric complications during delivery	Yes	115	28.6
	No	287	71.4
Intention to have more children	Yes	340	84.6
	No	62	15.4
Future place of delivery (n = 340)	This facility	180	44.8
	Another facility	79	19.7
	Home	81	20.1

**Fig 1:**
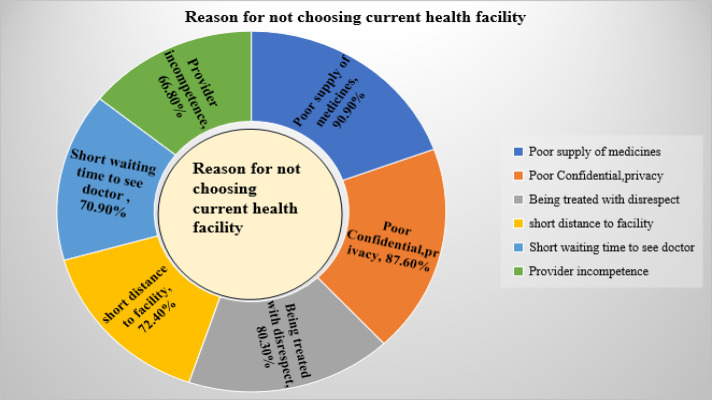
Reasons for not choosing current health facility by the women in Jimma Zone public hospitals, South West Ethiopia, 2023 (n = 402).

Among women who hadn’t had the intention to deliver in the health facilities, their main reasons for not delivering at health facilities in the prospective birth were poor supply of medicine, poor confidentiality and privacy that the women experienced, and being treated with disrespect during labour and childbirth (Fig 1).

### Knowledge of women’s rights during childbirth

The knowledge of women’s right is a fundamental tool to reduce OV and, promote respectful and dignified care during facility childbirth. The overall mean knowledge of the women towards their universal rights during childbirth was 14.89 ± 6. 76. This suggests that women’s knowledge is a significant concern within the group, requiring attention or intervention. Similarly, the current study highlighted that right to privacy and confidentiality (2.06 ± 1.39), right to liberty and freedom from coercion (1.72 ± 1.25), and right to be treated with dignity and respect (1.92 ± 1.20) have low mean scores with respect to other rights, indicating low women’s knowledge towards those rights (Table 4).

**Table 4:** Mean scores of women’s knowledge towards their rights during childbirth in public hospitals of Southwest Ethiopia, 2023.

Knowledge variables	Mean score
Right to information, informed consent and refusal, and respect for choices and preferences, including companionship during maternity care (4 items)	2.29 ± 1.42
Freedom from harm and ill treatment (3 items)	2.29 ± 1.18
Right to privacy and confidentiality (3 items)	2.06 ± 1.39
Right to be treated with dignity and respect (3 items)	1.92 ± 1.20
Right to equality, freedom from discrimination, and equitable care (3 items)	2.28 ± 1.24
Right to healthcare and to the highest attainable level of health (3 items)	2.33 ± 1.19
Right to liberty and freedom from coercion (3 items)	1.72 ± 1.25
**Overall mean knowledge (22 items)**	14.89 ± 6.76

### Attitude of women towards facility childbirth

The attitude of the women towards the intrapartum care process can be influenced by the negative experience in the health facilities and, normalization of this experience. In this study, the mean score of women’s attitude towards OV was found to be 33.55 ± 4.91, which is averagely unfavorable. Sub elements of women’s attitude includes unable to be informed about alternative birth positions apart from lithotomy position at 4.75 ± 0.60, disallowed to have a companion during delivery at 4.20 ± 1.26, failed to explain about examination or procedures that are going to be performed on their body at 3.15 ± 1.43, not asking permission/consent before any procedure at 3.14 ± 1.46, not considering disrespectful treatment during childbirth as a violence at 3.05 ± 1.32, and dissatisfied with the care they received from healthcare providers at this facility with the mean scores of 2.90 ± 1.00. These scores indicated that elements with higher mean values reflect a significant unfavorable attitude among women (Table 5).

**Table 5:** Mean distribution of women’s attitude towards facility childbirth in public hospitals of Southwest Ethiopia, 2023.

Attitude Variables	Mean scores
It is ok if healthcare providers discuss and share my private medical information to anyone	1.49 ± 0.85
It is not necessary to have Curtains, screens and linen to cover my body during labour and delivery	1.41 ± 0.75
Healthcare providers have the right to abuse me verbally	2.16 ± 1.37
Healthcare providers should not inform me about alternative birth positions apart from lithotomy position	4.75 ± 0.60
Disrespectful treatment during childbirth is not considered as a violence	3.05 ± 1.32
It is not a must to ask my permission/consent before any procedure	3.14 ± 1.46
Healthcare providers have no responsibility to explain about examination or procedures that are going to be performed on my body	3.15 ± 1.43
Physical abuse such as hitting, slapping, pushing, pinching, tied to delivery bed during childbirth is ok if I refuse/resist a healthcare provider	1.91 ± 1.24
Health care providers treat me poorly b/c of my background (sex, tribe, age, dress, ethnicity, religion/medical status Residence)	1.60 ± 1.13
Health care providers disallow me to have a companion during labour	2.09 ± 1.55
Health care providers have the right to disallow me to have a companion during delivery	4.20 ± 1.26
It is acceptable to retain me in the health facility if unable to pay my medical expense	1.59 ± 1.25
I dissatisfied with the care I received from healthcare providers at this facility	2.90 + 1.00
**Overall mean attitude (13 items)**	33.55 ± 4.91

### Status of OV

**Fig 2:**
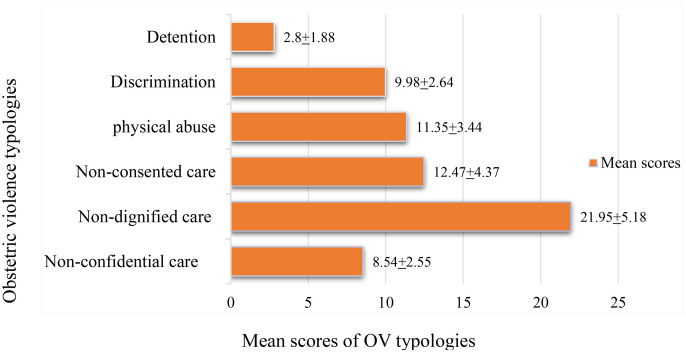
Mean distribution of obstetric violence typologies experienced by the women in Jimma Zone public hospitals, South West Ethiopia, 2023 (n = 402).

The study found that all participants experienced at least one form of OV, with an overall mean score of 67.09 ± 11.79 on a scale ranging from 40 to 104. The mean scores for specific types of OV were as follows: non-confidential care scored 8.54 ± 2.55, non-dignified care 21.95 ± 5.18, non-consented care 12.47 ± 4.37, physical abuse 11.35 ± 3.44, discrimination 9.98 ± 2.64, and detention 2.80 ± 1.88. Among these, non-dignified care was the most frequently reported type of obstetric violence, followed by non-consented care and physical abuse, while detention was reported the least (Fig 2).

### Factors associated with obstetric violence

On multivariable linear regression analysis, residence, occupation of the mothers, ANC contact, timing of birth, experience of obstetric complications and parity remained to have statistically significant association with obstetrics violence.

Women without obstetric complications had 8.07-point lower obstetric violence experiences compared to those with obstetric complications experiences (β = −8.07, 95%CI = −9.98, −6.16).

Each additional parity was associated with a 2.81- point lower obstetric violence score (β = −2.81, 95% CI = (−4.81–0.82).

Having two or more antenatal care (ANC) contacts and companion utilization during childbirth showed significant positive associations with women’s knowledge scores toward childbirth experiences and rights in the multivariable linear regression model. Specifically, each additional ANC contact was associated with a 3.23-point increase in knowledge scores (β = 3.23, 95% CI: 2.02, 4.45). Having companion during childbirth contributed an additional 4.87 points to women’s knowledge scores (β = 4.87, 95%CI = 2.14 to 7.59).

Regression analysis revealed factors statically significantly associated with women’s attitude and found that women residing in urban areas were associated with a 1.98-point decrease in unfavorable attitude (β = −1.89, 95% CI = −2.86, −1.01).

Women delivering at night time scored an average of 1.95 points higher OV than those in the daytime (β = 1.95, 95% CI = 1.65, 2.20).

Women without obstetric complications showed a 3.06-point decrease in unfavorable attitude scores compared to those with complications (β = −3.06, 95% CI: (−4.00, −2.11).

A one-unit increase in ANC contact was associated with a 3.36- point decrease in unfavorable attitude (β = −3.36, 95% CI = −4.13, −2.59) (Table 6).

**Table 6:** Multivariable Linear regression analysis factors for women’s knowledge, attitude and OV experiences in Jimma Zone public hospitals, Southwest Ethiopia, 2023.

	Obstetric violence			Women’s knowledge			Women’s attitude		
Variables	Unstandardized Adjusted(β) Coefficient	95% CI for β	P-value	Unstandardized Adjusted β	95% CI for β	P-value	Unstandardized Adjusted(β) Coefficient	95.0% CI for β	P-value
Age of the mother	0.05	(−0.14 0.25)	0.586	0.14	(−0.02 0.29)	0.09	–	–	–
Residence(urban)	−4.69	(−6.41 -2.96)	<0.001	**1.09**	(−0.29 2.47)	0.12	−1.89	(−2.86 -1.01)	**<0.001**
Educational status	0.82	(−0.19 1.83)	0.110	0.21	(−0.55 0.97)	0.59	0.14	(−.034 0.62)	0.568
Occupational status of the women (employed)	−1.38	(−2.08 -0.69)	<0.001	–	–	–	–	–	–
Wealth of the women (Second and above)	−0.25	(−0.80 0.30)	0.374	–	–	–	–	–	–
Sex of health care provider conducted delivery (female)	0.98	(−0.59 2.55)	0.221	–	–	–	–	–	–
Timing of birth(night)	4.06	(2.42 5.70)	<0.001	**−1.10**	(−2.40 0.21)	0.**10**	1.95	(1.65 2.20)	**0.022**
Mother’s Experience of complication during or after delivery (No)	−8.07	(−9.98 -6.16)	<0.001	**1.39**	(−0.14 2.91)	0.07	−3.06	(−4.00 -2.11)	**<0.001**
ANC contact (≥ 2)	−9.65	(−11.18 -8.11)	<0.001	3.23	(2.02 4.45)	**<0.001**	−3.36	(−4.13 -2.59)	**<0.001**
Parity (≥ 2)	−2.81	(−4.81 -0.82)	0.006	0.81	(−0.79 2.40)	0.32	−0.66	(−1.47 0.16	0.117
Companion utilization(yes)	−1.15	(−4.55 2.25)	0.506	**4.87**	(2.14 7.59)	**<0.001**	−1.77	(−3.45 -0.03)	**0.05**

Women living in urban areas had 4.69 points lower obstetric violence compared to those living in rural areas (β= (−4.69, 95%CI = −6.41–2.96).

Participants who are employee had 1.38 points lower obstetric violence compared to those unemployed (β = 1.38, 95%CI = 2.08, −0.69).

When the number of ANC contact was increased by one unit, the obstetrics violence that the women experienced decreased by 9.65points (β = −9.65, 95%CI = −11.18, −8.11).

Women delivering at night time scored an average of 4.06 points higher OV than those in the daytime (β = 4.06, 95%CI = 2.42, 5.70) (Table 6).

## Discussion

The present study assessed determinants of women’s knowledge, attitude and obstetric violence towards childbirth experience among women who gave childbirth in public health facilities of southwest Ethiopia. More than two-third (mean 14.89 SD 6.76, 68%) of women had adequate knowledge of childbirth experience particularly about childbirth rights, and half (mean 33.55 SD 4.91, 52%) and (mean 67.09 SD 11.79, 51%) of them had an unfavorable attitude and childbirth experience related to obstetric violence, respectively.

### Status of women’s knowledge, attitude and childbirth experience related to obstetric violence

Concerning the women’s knowledge about their rights during childbirth, in this study about 14.89 + 6.76 mean scores (68%) of women have good knowledge towards universal childbearing rights, which is lower. And the most common understood rights are information and informed consent, freedom from harm/ill-treatment, privacy/confidentiality, equality and equitable, healthcare and the highest attainable level of health. Similarly, literature reveals varying levels of women’s knowledge about their rights during childbirth, often described as fair or moderate in low-resource settings, with significant gaps contributing to obstetric violence and disrespectful care. In Nigeria, 76.9% of women attending a public hospital had fair knowledge of rights during pregnancy and childbirth [11]. This similarity may be attributed to the comparable measurement tools and methods employed across studies.

Women’s attitudes toward facility childbirth are often moderately negative, as evidenced by our study’s mean score of 33.55 (SD 4.91, 52%), shaped by fears of pain, complications, rights violations, and prior mistreatment, which heighten vulnerability to obstetric violence (OV), a pattern aligning with Ethiopian studies showing 41% home birth preference due to facility distrust and disrespectful treatment. Literature confirms that negative attitudes rooted in anxiety and trauma lead to poorer provider interactions and increased disrespect or abuse, whereas empowerment through rights education and ANC counseling fosters confidence, better communication, and reduced OV risk, shifting unfavorable views (e.g., from 68% to 6% tolerance post-intervention) [13,27,28].

Obstetric violence is a significant issue in maternal healthcare, particularly in Africa, where various studies have documented its prevalence and associated factors. The study revealed that all women who experienced at least one form of obstetric violence with the average score of 67.19 with the standard deviation of 11.79, indicating higher prevalence. The most prevalent form of OV was identified as non-dignified care, with a mean score of 21.95 ± 5.18. This was followed by non-consented care, which had a mean score of 12.47 ± 4.37. Similarly, A study conducted in Ethiopia reported that approximately 75.1% of women experienced some form of OV during labor and delivery, with non-consented care and non-dignified treatment being the most frequently reported forms [21]. Moreover, a study in Ghana found that the normalization of violence in healthcare settings contributed to a high prevalence of obstetric violence, with reports indicating that up to 83% of women experienced mistreatment during childbirth [12,29]. These findings highlight a troubling trend across different African contexts, where cultural acceptance and systemic failures in maternal healthcare contribute to the perpetuation of obstetric violence. In contrast, studies from other regions, such as Latin America, report varying prevalence rates of obstetric violence. For example, a study in Mexico indicated a prevalence rate of 33%, while Argentina reported 44% [12]. These differences may be attributed to cultural factors, healthcare system structures, and the varying degrees of awareness and reporting of obstetric violence.

### Determinates of women’s knowledge, attitude and childbirth experience related to obstetric violence

The present study shows OV has been reduced among women who had improved knowledge about their universal rights during childbirth. This is similar with other studies highlighting, the extent to which women are aware of their rights significantly influences their experiences of obstetric violence, with studies indicating that higher levels of awareness correlate with reduced instances of mistreatment [30,31]. This could be due to the women who are unaware of their rights may be more vulnerable to mistreatment, as they may not recognize abusive behaviors or feel empowered to challenge them [32].ANC contact is associated with increased women’s knowledge of childbirth rights, aligning with multiple studies on respectful maternity care (RMC) and antenatal education. Research consistently shows that women attending more ANC visits demonstrate better awareness of rights like informed consent, dignity, and freedom from abuse compared to those with fewer visits [33]. This is consistent with the present study, indicating participants who have frequent contact with healthcare providers have better knowledge about their rights compared to the counter parts. The possible justification could be methodological similarity. WHO emphasizes companions advocate for rights like informed consent and dignity by bridging communication gaps and witnessing care, indirectly reinforcing awareness during labour [34]. Similarly, In Ethiopia, low companion use correlates with poor rights knowledge. The current study also supports the statement of WHO, indicating those women who utilized the companions during labour and childbirth were increased level of knowledge towards birth rights. Every woman has a right to a companion of her choice to support her during labour and childbirth.

This study found that participants residing in urban areas, those without obstetric complications, women with more antenatal care contacts, and those utilizing companions during childbirth exhibited lower unfavorable attitudes toward birth experiences, whereas participants delivering at night showed higher unfavorable attitudes. This is consistent with the study conducted in Nigeria, Zambia and Karnataka, showing Urban residence was associated with lower unfavorable attitudes toward birth experiences (AOR: 0.42, 95% CI: 0.23–0.78), reflecting better access to information and services. Absence of obstetric complications correlated with reduced negative attitudes, as uncomplicated labors foster positive perceptions. Increased antenatal care contacts showed a strong inverse association, aligning with education-driven empowerment. Companion utilization during childbirth linked to lower unfavorable attitudes, enhancing support and rights advocacy. Nighttime deliveries, however, were associated with higher unfavorable attitude, likely due to rushed care and fatigue [11,33–35].

Research indicates that the prevalence and nature of OV can vary significantly based on the geographical location of the women receiving care. The current study indicated that women who reside in urban had decreased OV. This means those women who were from rural area had increased OV. This is in accordance with the study in Ghana highlighting, the overall prevalence of OV was high, the types of violence reported varied, with rural women more likely to experience neglect and disrespect during childbirth compared to their urban counterparts. This could be due to the normalization of violence in these context [29]. In this study, being employed decreased the incidence of OV. This is in line with the other Studies, indicating employed women are less likely to experience OV compared to those who are unemployed or housewives. For instance, findings suggest that women engaged in work are often more aware of their rights and more confident in expressing their needs during labor and delivery, leading to a reduced risk of experiencing mistreatment [21,36].

Interestingly, the current study showed that women who had increased ANC contacts had decreased obstetric violence. This finding seems contradict with the study in Ethiopia, showing women who had 4 or more ANC contacts had significantly higher odds of experiencing OV compared to those with fewer contacts [5]. This suggests that more frequent interaction with the healthcare system may increase exposure to potentially abusive or disrespectful treatment during childbirth. The general assumption that more prenatal care leads to better maternal and neonatal outcomes. Other studies in Ethiopia showed that increased ANC contacts were associated with improved prenatal care content [37] and higher likelihood of facility-based delivery [38]. The possible justification could be the study setting differences. The finding in the current study revealed that those women who delivered during the night time have increased incidence of OV. This finding was consistent with the study conducted in East Hararghe zone, reporting women who delivered during nighttime were more likely to report D&A compared to daytime deliveries [39,40]. This suggests nighttime deliveries may be associated with increased risk of OVin this region. However, a study in Dire Dawa city found that delivering at night was associated with lower likelihood of experiencing D&A compared to daytime deliveries [41]. This contradicts the findings from the current study and East Hararghe. The reasons for these conflicting results are not clear from the available information. Possible explanations could include differences in staffing levels, supervision, or facility practices between day and night shifts across different regions of Ethiopia. Obstetric complications are a widespread issue across multiple African countries, potentially contributing to or exacerbating instances of obstetric violence. In Ethiopia, research shows that women experiencing obstetric complications are more likely to report OV or D&A [5,42]. This is consistent with the current findings stating, women with obstetric complications had increased OV than women without complications. The possible justification would be this group of population need intensive care and continuous follow up which increases the burden on healthcare providers and also the duration they stay in the health facility are longer than other women. Thus, this may open the room that the women to experience obstetric violence.

### Implications of the study

This research documents the prevalence and determinants of obstetric violence in Jimma and surrounding areas, enabling health facilities to implement respectful maternity care. Improved provider knowledge reduces mistreatment incidence, enhancing maternal satisfaction, facility-based delivery uptake, and maternal-newborn outcomes, restores trust in health systems, and advances gender equality in healthcare in line with Ethiopia’s Health Sector Transformation Plan II., Findings guide the Ministry of Health and Regional Health Bureaus to prioritize high-risk factors (e.g., inadequate training, socio-cultural barriers) through community awareness campaigns, standardized protocols, and accountability mechanisms. Evidence supports integrating obstetric violence prevention into the upcoming Demographic Health Survey and national maternal health guidelines.

### Limitations of the study

Obstetric violence studies commonly face methodological challenges that limit generalizability and depth of insights. These include reliance on self-reported data prone to social desirability effects, inconsistent terminology and definitions across contexts, and predominant focus on women’s retrospective accounts over provider perspectives or prospective designs. In Ethiopia-specific research, cross-sectional approaches restrict causal inferences, while the relatively small sample size and two-facility selection limit generalizability beyond the study setting. Due to the sensitive nature of obstetric violence and its normalization, women may underreport incidents experienced during childbirth. Structural and, limited provider viewpoints and qualitative depth fail to capture enabling factors like workload and working environment. Thus, the future research should explicitly examine structural (facility), provider, and community factors independently using advanced methodology to better understand their distinct contributions to women’s knowledge, attitude and obstetric violence practices. Describing the physical childbirth environment helps clarify whether issues stem from providers, facilities, or both, without excusing any gaps in privacy or respectful care.

## Conclusion

The study demonstrated that more than two-thirds of participants relatively had a lower knowledge towards childbirth rights compared to other African countries. Similarly, around two- thirds of women held unfavorable attitudes and had experienced obstetric violence during childbirth. The most common types of violence reported were non-dignified care, non-consented care and physical abuse while detention was the least reported. Sociodemographic factors (residence and maternal occupation) and maternal factors (ANC contacts, birth timing, obstetric complications, companion utilization, and parity) predicted women’s knowledge, attitudes, and experiences of obstetric violence (OV) during childbirth. Therefore, it is essential to implement comprehensive training programs for healthcare providers that emphasize respectful and dignified care during childbirth. Additionally, enhancing women’s awareness of their rights related to maternity care, and improving women’s attitude through education initiatives can empower them to advocate for themselves and reduce instances of obstetric violence. Finally, Enhance ANC access, companion policies, and targeted strategies for high-risk groups (e.g., rural residents, certain occupations, emergency births) to create supportive childbirth environments, per public health guidelines emphasizing policy-practice alignment.
